# Yield of regular practice of high precordial leads electrocardiogram among asymptomatic upper Egyptian population considering Brugada patterns, a cross-sectional study

**DOI:** 10.1186/s43044-026-00714-x

**Published:** 2026-01-12

**Authors:** Alaa Rashad Ali, Mahmoud Abdelsabour, Salah Atta

**Affiliations:** Department of Cardiovascular Medicine, Assiut University Hospitals, Assiut, Egypt

**Keywords:** Brugada syndrome, High precordial leads, Prevalence, Brugada phenocopies, Arrhythmia

## Abstract

**Background:**

This cross-sectional study with limited prospective cohort follow-up aimed to determine the prevalence of Brugada-type ECG patterns (BTEPs) among asymptomatic Upper Egyptians using standard and high precordial leads, and to assess the short-term arrhythmic outcomes of positive cases.

**Methods:**

A total of 318 participants without arrhythmic symptoms were enrolled between June 2022 and June 2023. Standard 12-lead and high precordial ECGs (V1–V2 at 2nd intercostal space) were recorded. BTEPs were identified based on β-angle ≥ 58°, base length ≥ 1.5 mm at isoelectric line, and base width ≥ 4 mm at 0.5 mV. Positive cases underwent clinical follow up, echocardiography and Holter monitoring every 6 months for 12 months. Data was analyzed using McNemar’s test for paired proportions and logistic regression for confounder adjustment.

**Results:**

Two cases (0.63%; 95%CI: 0.17–2.26%) exhibited BTEPs on standard ECGs versus eight (2.52%; 95% CI:1.28–4.88%) on high precordial ECGs (*p* = 0.041). All positive cases were males. During follow-up, arrhythmias were documented in 7/8 cases (87.5%), including supraventricular tachycardia (*n* = 4), non-sustained VT (*n* = 1), and Mobitz I AV block (*n* = 1). No patient had family history of sudden cardiac death. The observed prevalence and arrhythmic rates were consistent with prior international data.

**Conclusion:**

Routine use of High precordial leads ECG in upper Egyptians showed comparable benefit of detecting Brugada patterns as reported globally. In addition to type I, the follow up of originally asymptomatic type II and III Brugada patterns may show variable arrhythmic presentations. However, due to small sample size and short-term follow-up, results should be considered preliminary pending larger confirmatory studies.

*Trial Registration* Our study has been registered as a clinical trial, clinicalTrial.gov ID: NCT05116488 at 10th November 2021.

## Introduction

Brugada syndrome (BrS) is an autosomal dominant arrhythmic disorder caused by abnormalities in cardiac ion channels, mainly sodium channels. It is potentially life-threatening due to ventricular tachyarrhythmia that can culminate in sudden cardiac death [1]. The description of the syndrome goes back to 1992. Pedro and Josep Brugada described seven patients suffering from persistent ST-segment elevation in leads V1-V3 who had survived sudden cardiac death due to polymorphic ventricular tachycardia without obvious structural heart disease [2].

Brugada syndrome accounts for 20% of sudden cardiac deaths from structurally normal hearts and 4% of overall cardiac-related deaths [3]. It has an estimated prevalence of 0.05% and it tends to be highest in Southeast Asia [4, 5]. However, many remain asymptomatic and are discovered only incidentally. Types II and III Brugada ECG patterns, which have lower risk for ventricular arrhythmias, frequently escape diagnosis due to their dynamic characteristics and because routine ECGs rather than more-sensitive high precordial leads are used [6, 7].

Brugada syndrome has 3 types of ECG patterns [8]; type I has coved type ST segment elevation of at least 2 mm with inverted T wave, type II has saddle-shaped ST segment elevation greater than or equal to 1 mm with biphasic T wave or positive T wave and type III which has the same features as Type I or II but with ST-segment elevation of < 1 mm.

Numerous studies indicated that the high precordial leads for the identification of Brugada patterns are more sensitive, especially with or without sodium channel blocker testing [9, 10]. In studies from Korea [11],Turkey [12], and the USA [13]. This method was used to evaluate prevalence; yet data on the Upper Egyptian population are scant. In part, this study attempts to fill that gap by evaluating the prevalence of Brugada ECG patterns identified by high precordial leads in an asymptomatic Upper Egyptian population.

## Methods

### Study population

The study involved 318 patients recruited between June 2022 and June 2023 at Assiut Cardiology Hospital.

Inclusion criteria: Patients of both genders, above 18 years old, presenting consecutively to our center during the study period, with non-arrhythmic symptoms nor indication.

Exclusion criteria: Patients with structural heart diseases including acute coronary syndrome, cardiomyopathies, severe valvular diseases, and hypertension with left ventricular hypertrophy were excluded from the investigation. Further exclusions included subjects with severe central nervous system, respiratory, endocrine, renal disorders, or electrolyte imbalances.

### Study design

Our study is a cross-sectional observational study with limited prospective cohort follow-up of positive cases. Both standard and high precordial ECGs (V1 and V2 moved to 2nd intercostal spaces) were used for the detection of Brugada patterns and their prevalence. Detailed history was re-taken for each positive patient, regarding syncope, tachycardia, and family history of sudden cardiac death. Positive cases were subjected to echocardiography, electrolytes, and cardiac enzymes analysis to rule out phenocopies to BTEPs. Positive Patients were counseled about their disease, the need to promptly correct fever, avoid electrolyte disturbance, and drugs which were known to aggravate the Brugada pattern (as specified on brugadadrugs.org).

The BTEPs were selected according to the following criteria to differentiate benign Rsrʹ patterns from BTEPs: β angle ≥ 58°, base length ≥ 1.5 mm at isoelectric line, and base width ≥ 4 mm at 0.5 mV distance from the triangle apex in leads V1 and V2 (the angle and the triangle formed by up and down slope of the rʹ wave). Presence of any of these criteria is considered diagnostic of BTEPs. The patterns were assessed by two investigators, discrepancies resolved by consensus, and inter-observer agreement assessed (κ = 0.86), showing the two reviewers had high level of agreement. In case of disagreement, a third independent reviewer was consulted.

Follow-up for each of the positive cases was done for a total of 12 months. This included clinical examination, ECG and ambulatory ECG (48-hour Holter monitoring) at baseline and six-month intervals to identify any clinical or subclinical arrhythmia. Holter recordings were interpreted by two electrophysiologists who were blinded to the baseline ECGs. Arrhythmias were defined according to current ESC criteria for definition.

### Sample size estimation

This study aimed to compare the detection of Brugada patterns using standard versus high precordial lead ECGs. With an expected detection rate of 0.6% as standard worldwide prevalence of type II, III Brugada patterns by standard ECG [4] and no fixed estimate for high precordial lead ECG due to limited prior data, we calculated the sample size using McNemar’s test for paired proportions. To detect a clinically relevant difference of 1.5%–2% with 80% power at a 5% significance level, a sample of 310–530 participants was required. The final sample of 318 subjects was adequate for this purpose.

### Ethical considerations

Approval was given by the Faculty of Medicine Ethics Committee, Assiut University (Approval date: 13th January 2022; IRB No: 17101610). Our study was conducted in accordance with the Declaration of Helsinki. Informed consent was obtained from all participants; all patients were informed about the study strategy and benefits.

### Trial registration

Our study has been registered as a clinical trial, clinicalTrial.gov ID: NCT05116488 at 10th November 2021.

Study had no financial funding from any source.

#### Statistical analysis

Basically, IBM SPSS A statistical software was used to analyze data (version 24.0). The descriptive statistics obtained were means, medians, standard deviations, and percentages. Group differences were tested using the Chi-square and Fisher Exact tests. Normality of data was tested using the Shapiro-wilk test. Continuous variables represented as mean ± SD (parametric) or, median [IQR] (non-parametric). Paired proportions (standard versus high ECG) were compared using McNemar’s test. Depending on data distribution, Student’s t-test and Mann-Whitney were used for parametric and non-parametric group comparisons, respectively. A p-value < 0.05 was considered statistically significant. Age and sex confounding effects were considered in a logistic regression.

## Results

**Fig. 1:**
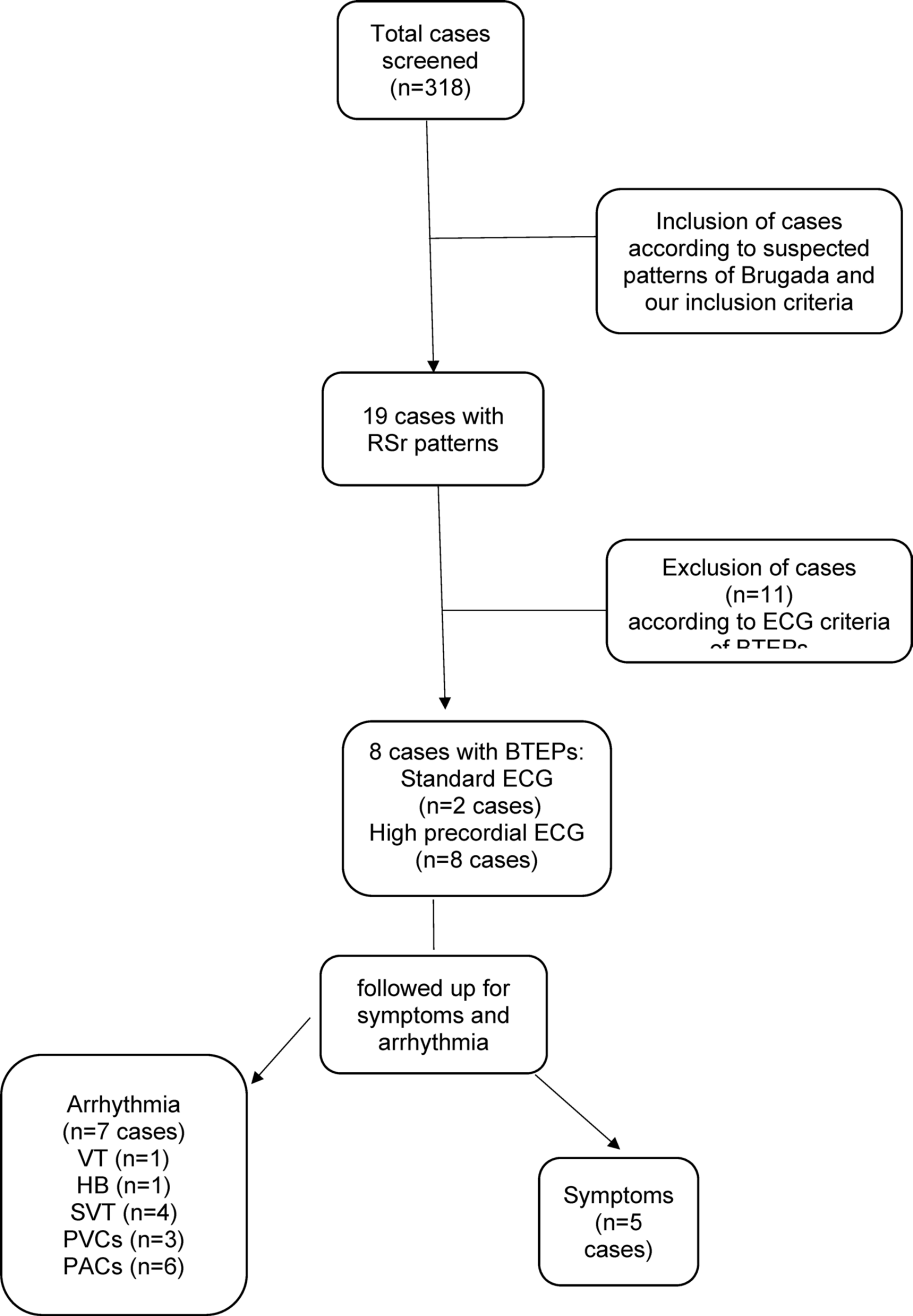
Flow chart of the study cases and positive data,* BTEPs* Brugada-type ECG patterns,* HB* heart block,* NCT* narrow complex tachyarrhythmias,* PACs* premature atrial contractions,* PVCs* premature ventricular contractions,* SVT* supraventricular tachycardia,* VT* ventricular tachycardia

The study included 318 patients including 189 males (59.4%). The mean age was 45.27 ± 15.2 years after applying the exclusion criteria. 19 patients had RSr’ pattern, among them 11 patients not fulfilling the specific diagnostic criteria were excluded. By using standard ECG, 2 cases (0.63%; 95%CI:0.17–2.26%) showed Brugada-type ECG patterns, and by using high precordial ECG in the 2nd intercostal space, 8 cases (2.52%; 95%CI:1.28–4.88%) were detected. The prevalence difference between the standard and high precordial ECG was statistically significant (*p* = 0.041). (Fig. 1)

Out of the eight positive cases, there were four cases with type II, three with type III, and one with type I Brugada pattern. In their standard ECG, one case showed spontaneous type I, one had type III, two were normal, two had inverted T in V1, and two had rSr′ pattern in V1.

The eight positive cases for Brugada ECG patterns were males and had normal Echocardiography and electrolytes. During follow-up, two patients had a history of tachy-palpitation and syncope, three had episodes of tachy-palpitation only, the other three patients were asymptomatic but one of them exhibited significant arrhythmias during follow-up Holter monitoring. No one had a family history of sudden cardiac death. (Tables 1 and 2)

**Table 1:** Brugada-type ECG pattern (BTEP) at 2nd (High) ^/^4th (Standard) intercostal spaces of the 8 positive patients

BTEP	Standard ECG	High ECG
Type I	1 (0.3%)	1 (0.3%)
Type II	0	4 (1.3%)
Type III	1 (0.3%)	3 (0.9%)
Total	2 (0.6%)	8 (2.5%)

**Table 2:** The positive cases’ arranged by age and ECG pattern

SN	Age/year	Standard ECG	High ECG	Symptoms	Arrhythmia	Arrhythmia ESC class
1	18	Normal	Brugada pattern type III	No	No	No
2	30	Normal - RSr pattern V1	Brugada pattern type II	Tachypalpitations /Syncope	2nd HB	Mobitz type I and 2:1 AV block
3	31	Normal - RSr pattern V1	Brugada pattern type II	No	PACs	NCT
4	36	Normal -Inverted T V1	Brugada pattern type III	Tachypalpitation	PACs	NCT
5	38	Normal - Inverted T V1	Brugada pattern type II	Tachypalpitation	SVT/ PACs	NCT
6	48	Spontaneous Brugada Type I	Spontaneous Brugada Type I	Tachypalpitation	Non-sustained VT/ SVT/Ventricular Bigeminy/PACs	Non-sustained VT Short -coupled PVCs NCT
7	62	Normal	Brugada pattern type III	No	SVT/PACs/PVCs	NCT Monomorphic PVCs
8	67	Brugada pattern type III	Brugada pattern type II	Tachypalpitations/Syncope	SVT/Ventricular Bigeminy/ PACs	Short- coupled PVCs NCT

*AV block* Atrio-ventricular block,* NCT* narrow complex tachyarrhythmias,* PACs* premature atrial contractions,* PVCs* premature ventricular contractions,* SVT* supraventricular tachycardia,* VT* ventricular tachycardia

Ambulatory ECG (Holter) monitoring revealed the following arrhythmias: Supraventricular Tachycardia (SVT) in 4 cases (50%), Non-Sustained Ventricular Tachycardia (VT) in 1 case (12.5%) with polymorphic morphology and high-risk features, Premature Atrial Complexes (PACs) in6 cases (75%) and Premature Ventricular Complexes (PVCs) in 3 cases (37.5%) with 1 case had monomorphic PVCs and 2 cases had polymorphic PVCs with high-risk features (R-on-T phenomenon, frequent bigeminy). Also, Heart Block (2nd Degree) was recorded in 1 case (12.5%) in the form of Mobitz type I (Wenckebach) and occasional 2:1 AV block. Nearly all patients with Brugada ECG patterns in our study had some sort of arrhythmia that may contribute to their symptoms and may be related to a degree of channelopathy. (Tables 1 and 2)

**Fig. 2:**
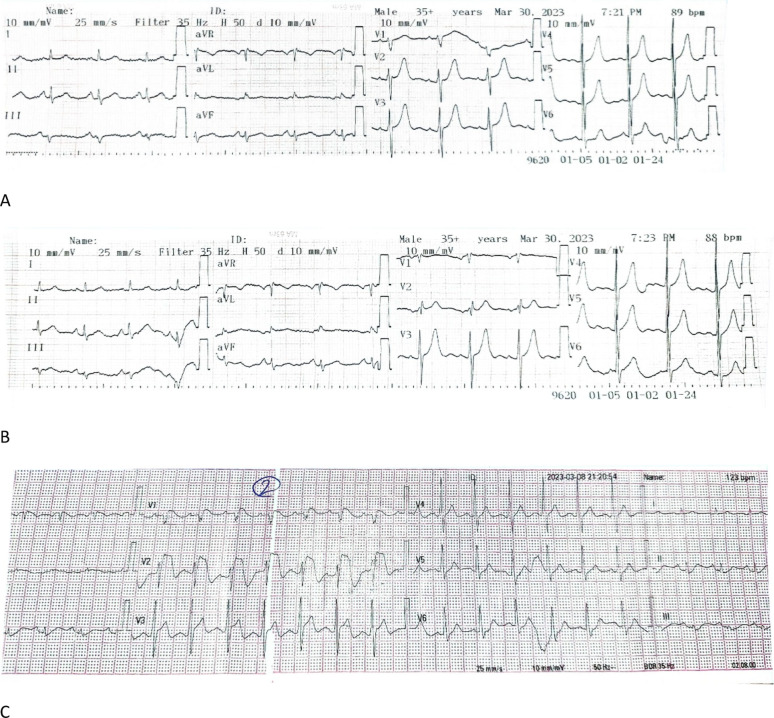

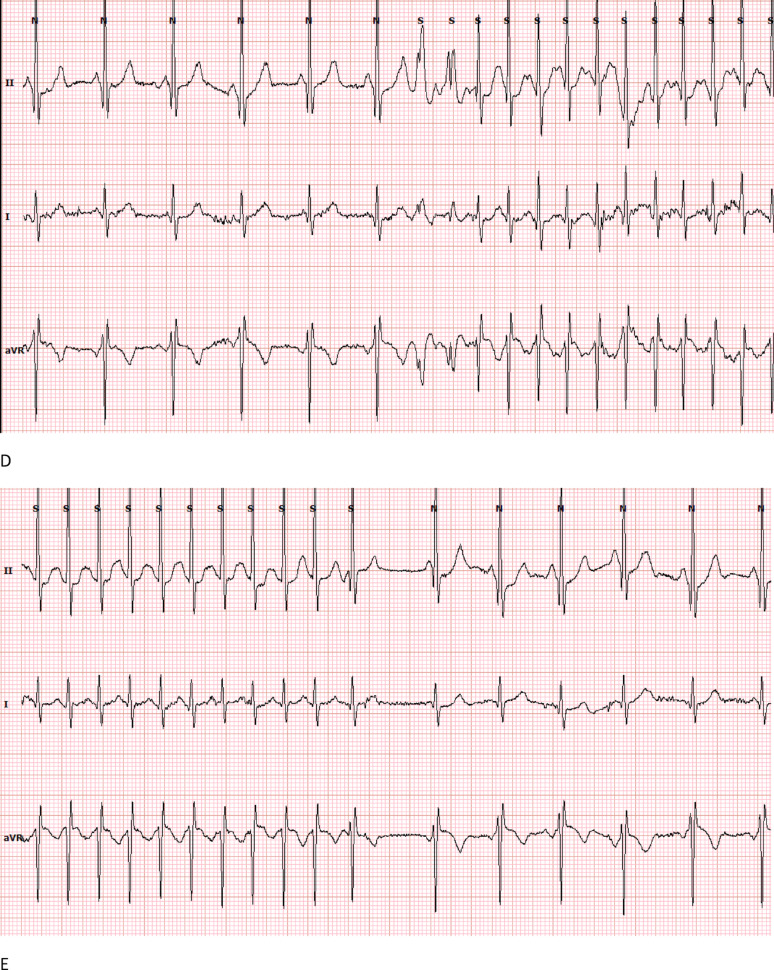
ECGs and arrhythmia detected in Holter of Case Number 7 (**A**: standard ECG/** B**: High ECG/**C**: ECG during fever/** D** and **E**: SVT attack onset and offset)

During follow up, Dynamic Changes of Brugada Patterns were recorded; One case exhibited Type I Brugada pattern during fever (pneumonia), which reverted to Type III upon resolution of fever and two cases showed alternating Brugada patterns Type II in high ECG and normal standard ECG, later showing Type II in standard ECG. (Fig. 2)

We also detected two Brugada phenocopies who were excluded; One presented with Type III in high ECG but was diagnosed as ischemic heart disease through echocardiography and coronary angiography. The other case showed Type I in high ECG but Type II in standard ECG and also had ischemic etiology.

When applying the ECG criteria for selecting Brugada patterns (β-angle, the length of the triangle base at the isoelectric line, and 0.5 mV), the following results were observed (Table 3): The average length of the triangle base at 0.5 mV in leads V1 and V2 was 2.94 ± 0.9 mm and 2.31 ± 0.9 mm, respectively. Four patients had a base length ≥ 4 mm in V1, and two patients met the same criterion in V2. The average length of the triangle base measured at the isoelectric line in leads V1 and V2 was 1.69 ± 0.6 mm and 1.56 ± 0.5 mm, respectively. Six patients met the criterion of ≥ 1.5 mm in both V1 and V2. The average β-angle measured in leads V1 and V2 was 33.13 ± 12.9° and 24.75 ± 8.5°, respectively. None of the patients met the criterion of a β-angle ≥ 58°. Overall, the selected cases met the criteria in V1 and/or V2 at the isoelectric line and/or at 0.5 mV.

**Table 3:** Diagnostic characteristics of positive cases (ECG Criteria)

SN	β-angle≥ 58°	Base of ∆ at Iso- electric Line	Base of ∆at 0.5mv	Number of positive criteria for each case
1	Negative	Negative	Positive	1
2	Negative	Positive	Negative	1
3	Negative	Positive	Negative	1
4	Negative	Negative	Positive	1
5	Negative	Positive	Positive	2
6	Negative	Positive	Positive	2
7	Negative	Positive	Negative	1
8	Negative	Positive	Negative	1

## Discussion

Our study is the 1st among upper Egyptian adult population subjecting such relatively large sample size to high precordial ECG recording. The BTEPs in high precordial leads was about 2.52% compared to 0.63% in standard 12-lead ECG. The exploratory risk ratio (RR) was approximately 4.0, showing that high precordial ECG can exploit an increased sensitivity in detecting Brugada patterns in asymptomatic patients with structurally normal hearts among upper Egyptians.

Among other studies, one reference study reported the percentage of Brugada Type II and III ECG at around 0.6% with standard ECG and noted that in the Middle East it reached 1% [4]. Compared with the global logs; our present study matched those findings using standard ECG but showed some higher numbers using high precordial ECG. Our exploratory RR was consistent with other available reports from Turkey, Korea, and the USA. For instance, a study from Turkey [12] noted a rise in detection rates from 2.97% in standard ECG to 7.54% with high precordial ECG at the 2nd intercostal space (RR of approximately 2.5). Yet again, in a Korean study [11], Brugada patterns were observed in 1.3% of healthy individuals based on high precordial ECG in comparison to standard ECG with none identified.

The increase in the functional stability of high precordial ECG and its closer relation to right ventricular outflow tract area induces considerably greater sensitivity, leading to a much more robust determination of prevalence. It might prove especially useful in parts of the world where Brugada syndrome prevalence is limited, as in Upper Egypt.

The ECG criteria used in this present study to identify Brugada ECG patterns were recommended by several studies. Those included β-angle ≥ 58°, length of the triangle base at the isoelectric line ≥ 1.5 mm, and at 0.5 mV ≥ 4 mm, which helped in the differentiation of Brugada ECG patterns from the benign RSr’ patterns. Certainly, the adopted criteria influence the sensitivity and rate of detecting positive cases. For instance, Peritz and Chung [9], as well as Chevallier et al. [14] and Ohkubo et al. [15], described the β-angle criterion with a cutoff value of ≥ 58°.

Nevertheless, two other studies by Serra et al. and Carrington et al. [16, 17] used similar cutoff values but set the lower threshold for the β-angle at ≥ 36.8°. Thus, if we had used this cutoff value in our study, the β-angle criterion in lead V1 would have been met in three more cases. Also, Vetta et al. [18] proposed different cutoff criteria: β-angle ≥ 40°, length of triangle base at the isoelectric line ≥ 2 mm, length of triangle base at 0.5 mV ≥ 3 mm. If we applied these criteria in our study: Three more cases in lead V1 would have been classified as having the β-angle ≥ 40°, in lead V1, three additional cases would have had a base length of the triangle at the isoelectric line of at least 2 mm, while two did so in lead V2. Five additional cases would meet the criterion of a triangle-base length at 0.5 mV of at least 3 mm in lead V1, while two did so in lead V2. Such findings expose that the variability of results might be according to the criteria set, methodological and population characteristic differences across studies.

In view of the arrhythmic risk in Non-Type I BTEP, this study illustrates the arrhythmic potential in non-Type I Brugada patterns. Eight originally asymptomatic cases were evaluated; five of them developed arrhythmias, one had Type I BTEP and four had either Type II or III. The arrhythmias noted during follow-up included SVT, non-sustained VT, PVCs and 2nd-degree AV block, realizing the need for great care for follow-ups in non-Type I cases.

Dynamic ECG changes in two cases further demonstrate the necessity of high precordial leads for accurate diagnosis and monitoring. Fever-induced Type I patterns, as observed in one patient, align with known Brugada syndrome triggers, reinforcing the importance of fever management in these individuals.

Short-term Holter monitoring revealed significant arrhythmias, including high-risk polymorphic VT, PVCs, SVT and second-degree AV block. These findings align with previous studies on Brugada syndrome, suggesting that sodium channel dysfunction affects not only ventricular cells but also atrial and AV nodal tissues [19, 20].

Our findings underscore the importance of follow-up for asymptomatic BTEP cases, including non-Type I patterns, due to their potential arrhythmic risks. Fever management and avoidance of triggering medications are critical for patient safety. Further research with larger cohorts and long-term monitoring is warranted to refine risk stratification and management strategies for Brugada syndrome.

### Study limitations

Our study detected higher sensitivity of high precordial ECG and significant arrhythmic events in positive cases, but the small sample of positive cases and the short-term follow-up applied only to the positive cases, limit the value of our results, and necessitates larger comparative studies to confirm the results. The study population was recruited from a tertiary-care hospital, the external validity to the general Upper Egyptian population may be limited. The potential Selection bias, observer bias, and follow-up bias were decreased by standardized criteria and blinded Holter ECG readings, yet larger studies involving the general population are recommended for more generalizability of our study results. The non availability of pharmacological challenge testing (e.g., ajmaline/flecainide) may also affect the diagnostic sensitivity of our results.

## Conclusion

The routine use of High precordial leads ECG in upper Egyptians showed comparable benefit of detecting Brugada patterns as reported globally. In addition to type I, the follow up of originally asymptomatic type II and III Brugada patterns may show variable arrhythmic presentations. However, due to small sample size and short-term follow-up, results should be considered preliminary pending larger confirmatory studies.

## Data Availability

Data is provided within the manuscript.

## Abbreviations

BrS: Brugada syndrome
BTEPs: Brugada type ECG patterns
ECG: Electrocardiogram
HB: Heart block
PACs: Premature atrial complexes
PVCs: Premature ventricular complexes
SVT: Supraventricular tachycardia
VT: Ventricular tachycardia
